# Area of focus to handle delays related to maternal death in Ethiopia

**DOI:** 10.1371/journal.pone.0274909

**Published:** 2022-09-19

**Authors:** Neamin Tesfay, Rozina Tariku, Alemu Zenebe, Fetiya Mohammed, Fitsum Woldeyohannes

**Affiliations:** 1 Center of Public Health Emergency Management, Ethiopian Public Health Institutes, Addis Ababa, Ethiopia; 2 Health Financing Program, Clinton Health Access Initiative, Addis Ababa, Ethiopia; Aga Khan University pakistan, PAKISTAN

## Abstract

**Background:**

Maternal delay factors, together with medical factors, have a substantial role in determining maternity outcomes. Although several studies were conducted on delay factors that contribute to maternal death in Ethiopia, the studies were mostly focused either on an individual or at a provincial level factor with a limited number of study participants. In response to this gap, this study is aimed at exploring the magnitude and factors related to delay factors that contribute to maternal death in Ethiopia.

**Methods:**

The study used maternal death surveillance data collected from different regions of Ethiopia, compiled between 2013 and 2021. A total of 4530 maternal deaths were reviewed during the study period. A Multilevel multinomial logistic regression model was applied to examine factors associated with delays related to maternal death. An adjusted relative risk ratio with a 95% confidence interval was stated and variables with p-values less than 0.05 were declared as significant predictors of maternal delay.

**Result:**

Delay three (delay in receiving adequate and appropriate care once reached a health facility) has contributed to 36.3% of maternal deaths followed by delay one (delay in deciding to seek care when experiencing an obstetric emergency) and delay two (delay in reaching to an appropriate obstetric facility) where each of them contributed to 36.1% and 27.6% of maternal deaths respectively. In the multivariate multilevel multinomial model, maternal age, education status, and place of death were among the individual level factors associated with both delay two and delay three. Conversely, marital status and ANC follow-up were associated with delay two alone, while the timing of maternal death was associated with delay three. Residence and type of facility were provincial-level factors linked with both delay two and delay three, while the type of region was related to delay three of maternal death.

**Conclusion:**

Both delay one and three have a major contribution to maternal death in Ethiopia. Individual and provincial level factors played an important role in determining delays related to maternal death. Therefore, it is crucial to account for measures that provide emphasis on the area of raising awareness on the utilization of Antenatal care (ANC) service, improving facility readiness to handle obstetrics emergencies, and narrowing down inequality among regions in service provision.

## Introduction

Maternal death is one of the commonly used yardsticks to assess the performance of health systems; it is also an important proxy parameter to measure women’s empowerment and national development [1]. Globally, the burden of maternal death is more prominent in Sub-Saharan African and South Asian countries [2]. Specifically, Sub-Saharan African countries accounted for nearly 67% of global maternal deaths in 2017 [3]. To address these challenges global target was set in 2000, called the millennium development goal (MDG) which lasted fifteen years. To keep the momentum generated by MDG, a new global target was established in 2015 under the sustainable development goals (SDG). The SDG has a target to reduce maternal death to as few as 70 deaths per 100,000 live births by 2030 [4].

Ethiopia is one of the nations that has reduced maternal death remarkably in the last two decades. According to the Ethiopian Demographic and Health Survey (EDHS), there was a substantial decline in the maternal mortality rate, from 871 death per 100,000 live births in 2000 to 412 deaths per 100,000 live births in 2016 [5]. Despite a tangible reduction in the overall maternal mortality rate, the presence of a noticeable regional variation was one of the factors that have hampered the achievement of the target set under the MDGs [6, 7]. To better streamline the information flow on maternal mortality, Ethiopia has established the maternal death surveillance and response system (MDSR) in 2013, which is aimed at monitoring the progress towards the goals of MDG [8].

MDSR is an ongoing system employed for the systematic identification, collection, review, analysis, and interpretation of data related to maternal death [9]. The surveillance system is accompanied by a response component, which is implemented at individual and national levels to avert similar death in the future [10]. Countries like Ethiopia, which have a weak vital registration system in place and a low rate of institutional delivery are recommended to practice a mix of facility-based and community-based reviews of maternal death to ensure the coverage and completeness of the system [11]. Currently, Ethiopia is practicing this mixed method of registering maternal death [12], while its implementation is being challenged by underreporting, limited capacity of the health workforce, and low engagement of the community [13, 14].

MDSR data can guide in identifying the area of interventions to tackle the delay factors related to maternal death and it enables to design individual-level responses at health facility level and programmatic responses at the national and sub-national levels [15, 16]. The three-delay model was developed by Thaddeus and Maine in 1994 to facilitate the identification of indirect factors, from the onset of obstetric complications to the birth of the baby [17, 18]. Delay one is related to delay in deciding to seek care, which is commonly influenced by negative past experiences, late recognition of the problem, and financial problems [19]. Delay two, on the other hand, is related to identifying and reaching care, which is usually determined by the availability of transport and accessibility of the facility [20–22]. The third delay, i.e., delay three, is related to receiving suboptimal care after reaching a health facility [23], which is usually measured by the availability of trained personnel, essential medical commodities, and initiation of early treatment and follow-up [24].

According to the study by Azmach and Ftalew et al. [25], delay one contributes to 33% of maternal death followed by delay two and delay three, which contributes to 32% and 29% of maternal deaths in Ethiopia respectively. In line with this, per the 2019 annual report of the Ethiopian Ministry of Health, the contribution of delay one is declining over time and the lead is being taken by delay three [12].

Ethiopia has implemented various initiatives to handle delays related to maternal death such as the establishment of maternity waiting rooms in a health facility [26], availing free transport and maternity service [27], and the introduction of health extension and community-based health insurance (CBHI) programs [28, 29]. Moreover, middle-level health cadres (Integrated Emergency Surgical Officers) are being trained to handle obstetric emergencies at a lower level. This is coupled with upgrading the existing health facilities to ensure the provision of comprehensive emergency obstetrics and newborn care (CEmONC) [30, 31]. Despite all this effort, however, the number of maternal deaths remains unacceptably high [32].

Educational status, marital status, ANC service utilization, maternal parity, and place of delivery were individual-level factors that contribute to maternal delay whereas, the type of health facility, residence of the women, and type of region were provincial factors related to maternal delay [33–35]. However, none of the studies had used these factors (i.e., individual, and provincial factors) simultaneously. Furthermore, most of the studies were focused on small and localized areas with a limited study population and descriptive form of analysis. In response to this gap, this study is aimed at determining the magnitude and identifying factors associated with delay factors related to maternal death using multilevel analysis based on the MDSR data.

## Methods and materials

### Study setting

Ethiopia has an estimated population of 114,963,588 in 2020, out of which 28,807,161 were women of reproductive age group [36]. Administratively, Ethiopia has nine regions and two city administrations, namely Tigray, Afar, Amhara, Oromia, Somali, Benishangul-Gumuz, Southern Nations Nationalities and Peoples Region (SNNPR), Gambella, Harari, Addis Ababa city administration and Dire Dawa city administration [37]. The country has a total of 17187 health posts, 3724 health centers, 302 public hospitals, and 5401 private health facilities. The country has 0.44,0.97, 0.8, and 8.4 per 10000 population of medical doctors (including specialists), health officers, midwives, and nurses respectively [38].

### Study design and data collection process

A secondary data review was conducted on Ethiopian MDSR data gathered and compiled from all regions of the country. The data was collected through the MDSR system, which enforces a mandatory notification, investigation, and verification of maternal deaths across the country. Moreover, the system was accompanied by a review of each notified maternal death to assign the cause and contributing factors related to the death. The review process uses two data sources, namely: facility-based abstraction format (FBAF) for health facility deaths and verbal autopsy (VA) for community deaths. At a health facility level, a maternal death review is conducted based on the abstraction format, by the MDSR committee. After the death review, the final death report is preprepared using the maternal death reporting format (MDRF) by the assigned surveillance focal person. Afterward, the report is sent to the national data hub and this national data is used as a data source for this study [39]. A total of 4530 maternal deaths were reviewed from 2013 to 2021 in Ethiopia. The MDSR data is hierarchical i.e., women were nested in reporting facilities and reporting facilities were nested in different zones(provinces) of the country.

### Case definition and inclusion and exclusion criteria

#### Case definition

Maternal death is the death of a woman while pregnant or within 42 days of termination of pregnancy, irrespective of the duration and the site of the pregnancy, from any cause related to or aggravated by the pregnancy or its management, but not from accidental or incidental causes [39].

#### Inclusion criteria

Women who meet the case definition stated above were included in the study.

#### Exclusion criteria

Women who died due to an accidental or incidental cause during pregnancy or a within 42 days of termination of pregnancy were excluded from the study.

### Study variables

#### Outcome variables

The delay factor was used as the study’s principal outcome variable. It had three distinct categories; delay one (delay in seeking health care), delay two (delay in reaching a health care facility), and delay three (delay in receiving care at the health facility). The classification was made using 14 variables, which were used to assess the contributing factors to maternal deaths. Delay one was measured using 5 item questionnaires that include 1) visiting a traditional healer or traditional birth attendant first 2) the family having insufficient money 3) lack of awareness of obstetric complications 4) the nearest healthcare facility is more than 5 km away 5) lack of decision to health facility due to perceived poor quality of care at a health facility.

The second delay was assessed using 5 item questions that include 1) the poor road condition and/or terrain2) long travel time from home to a healthcare facility (more than an hour) 3) the cost of transportation 4) unavailability of transportation 5) unavailability of healthcare facility in the area (takes more than one hour to reach the nearest healthcare facility).

The third delay was also measured using 4 item question that includes: 1) inadequacy of the referral system, (ambulances not available, no fuel, breakdown, and use of public transport) 2) shortage of equipment and supplies 3) delayed management after admission (more than 30 min from the time of arrival to time of assessment or receiving treatment) 4) wrong assessment of risk, wrong diagnosis, and wrong treatment. Three procedures were followed to decide the significant delay factor that contributed to the death of each mother. First, the row sum score of each delay was computed and this was followed by standardization of scores, which rescale the scores to have a mean value of 0 and a standard deviation of 1, to compare the score of each delay based on the value of standard deviation [40]. Finally, the delay with the highest score of standard deviation was selected as the perceptible delay factor that contributes to maternal death.

#### Explanatory variables

Both individual and community(provincial) level variables were included as a predictor in the model. Individual-level variables includes women’s age, educational status, marital status, parity, history of Antenatal Care (ANC) follow-up, place of death, time of death, and cause of death. The medical cause of death was incorporated as individual death after the underlying cause of death was assigned using the International Classification of Diseases -Maternal Mortality (ICD-MM) [41].

Community(provincial) level variables include variables such as residence, type of facility, and type of region. The type of region was classified into three categories (city, agrarian, and pastoralist) based on the cultural and socio-economic backgrounds of the population [42]. Furthermore, the type of facility was codified into classes (primary, secondary, and tertiary facilities) according to their manpower, medical equipment, and service provision [38].

### Data management and statical analysis

The data was exported from Epi -info version 7.2 to Stata version 17 for data cleaning and further analysis. using the cleaned data, both descriptive (count and percentage) and analytical analysis (multilevel multinomial logistic regression) were carried out and reported.

#### Model building

Multilevel multinomial logistics regression was employed to incorporate random and fixed effects into the model. The analysis was performed using Generalized Structural Equation Modelling (with the logit link function) using the “gsem” Stata command [43]. While defining the model, delay one was selected as a reference category of the independent variable. Four consecutive models were fitted to decide on the final model. The first model was a null model (containing only the outcome variable), the second one was model 1 (model fitted using individual-level variables only), the third was model 2(model fitted using provincial-level variables only) and the fourth model was model 3 (fitted using individual and provincial variables). The final best-fitted model was selected based on the value of log-likelihood and Akaike’s information criteria (AIC). Both bivariate and multivariate analysis were employed. A P-value of less than 0.20 was used as a cut-off point to retain variables for the final multivariable analysis. Multicollinearity between explanatory variables was checked using the Variance Inflation Factor (VIF). Finally, the adjusted Relative Risk Ratio (RRR) with 95% Confidence Interval (CI) was reported and variables with p- values <0.05, in multivariate analysis, were declared as significant predictors of delay two and delay three.

In the random-effects analysis, to assess the variability of delay two/three between provinces, both Intraclass Correlation Coefficient (ICC) and Proportional change in Variance (PCV) were calculated.

#### Ethical statement

Ethical approval was obtained from the Ethical Review Committee and Public Health Emergency Management unit of Ethiopian Public Health Institute (EPHI) with Ref. No. EPHI 4_1/37. Since the study used secondary data, other ethical measures were not required.

## Result

### Selected characteristics of reported facilities

A total of 4530 maternal death were reviewed during the study period. Delay three has contributed to 36.3% of maternal deaths followed by delay one and delay two where each contributed to 36.1% and 27.6% of maternal death, respectively. Among the reported facilities, nearly half of the maternal death (44.3%) contributed by delay one was reported from the primary health care level. Similarly, more than half of maternal death (53.5%) contributed by delay three were reported from tertiary level health care. Region-wise, 42.3% of maternal death in the Afar region was contributed by delay one, whereas 29.8% of maternal deaths in the Amhara region were contributed by delay two. Furthermore, 55.2% of maternal death in the Somali region were contributed by delay three (Table 1).

**Table 1:** Selected background characteristics of reporting facilities by the delay factor in Ethiopia, 2021.

Variable/category	Delay factor			Total	Significant
	Delay one (n = 1635)	Delay two (n = 1251)	Delay three(n = 1644)		
	(%)	(%)	(%)		
**Type of health facility**					
Primary level health care	1154(44.3)	754(29.0)	696(26.7)	2604	0.001
Secondary-level health care	266(28.1)	257(27.1)	424(44.8)	947	
Tertiary level health care	215(22.0)	240(24.5)	524(53.5)	979	
**Ownership of facility**					
Private	1(8.3)	6(50.0)	5(41.7)	12	0.181
NGO	13(33.3)	14(35.9)	12(30.8)	39	
Government	1621(36.2)	1231(27.5)	1627(36.3)	4479	
**Reporting region**					0.424
Tigray	209(36.9)	138(24.4)	219(38.7)	566	
Afar	33(42.3)	18(23.1)	27(34.6)	78	
Amhara	431(34.5)	373(29.8)	446(35.7)	1250	
Oromia	516(36.6)	395(28.0)	498(35.3)	1409	
Somali	10(34.5)	3(10.3)	16(55.2)	29	
Ben-Gum	28(35.9)	22(28.2)	28(35.9)	78	
SNNPR	196(34.9)	157(27.9)	209(37.2)	562	
Gambella	12(37.5)	7(21.9)	13(40.6)	32	
Harari	28(32.2)	20(23.0)	39(44.8)	87	
Addis Ababa	108(39.4)	76(27.7)	90(32.9)	274	
Dire Dawa	64(38.8)	42(25.5)	59(35.8)	165	
**Year of reporting**					
2013	4(30.8)	7(53.9)	2(15.4)	13	0.001
2014	126(42.6)	70(23.7)	100(33.8)	296	
2015	219(44.6)	124(25.3)	148(30.1)	491	
2016	282(34.1)	251(30.4)	294(35.6)	827	
2017	462(35.5)	321(24.7)	518(39.8)	1301	
2018	243(35.6)	171(25.1)	268(39.3)	682	
2019	148(31.1)	162(34.0)	166(34.9)	476	
2020	151(34.0)	145(32.4)	148(33.3)	444	

### Sociodemographic characteristics of the deceased women

The proportion of women who died due to the contribution of delay one was higher among women who are in the age group of 10–19 years (42.6%) compared to women who were aged 20–29 years (35.4%). Women who resided in a rural area had a higher proportion of death due to delay three (47.9%) compared to those who resided in an urban area (34.2%). Likewise, women who lived in cities had a higher proportion of death due to the contribution of delay three (48.0%) compared to those who reside in the pastoralist area (31.3%). In line with this, women who attended secondary education and above had a higher proportion of death due to the influence of delay three (48.5%) compared to women who only attended primary level education (34.4%). Besides, women who died during the antepartum period had a higher proportion of death due to delay two (36.1%) compared to women who died during the postpartum period (26.8%) (Table 2).

**Table 2:** Distribution of personal characteristics of the deceased women by delay factor in Ethiopia, 2021.

Variable/Category	Delay factors			Total	Significant
	Delay one(n = 1635) (%)	Delay two(n = 1251) (%)	Delay three(n = 1644) (%)		
**Age group**					
10_19	110(42.6)	65(25.2)	83(32.2)	258	
20_29	781(35.4)	596(27.0)	827(37.5)	2204	
30_39	649(35.7)	507(27.9)	660(36.3)	1816	0.052
40_49	95(37.7)	83(32.9)	74(29.4)	252	
**Residence area**					
Urban	156(22.2)	210(29.9)	336(47.9)	702	0.001
Rural	1479(38.6)	1041(27.2)	1308(34.2)	3828	
**Regional residence**					
Emerging	86(39.6)	63(29.0)	68(31.3)	217	
City Administration	148(28.0)	127(24.0)	254(48.0)	529	0.001
Agrarian	1401(37.0)	1061(28.0)	1322(34.9)	3784	
**Place of death**					
On transit	209(34.2)	226(36.9)	177(28.9)	612	
Home	529(66.8)	188(23.7)	75(9.5)	792	0.001
Health facility	897(28.7)	837(26.8)	1392(44.5)	3126	
Marital Status					
Unmarried	124(42.2)	80(27.2)	90(30.6)	294	0.049
Married	1511(35.7)	1171(27.6)	1554(26.7)	4236	
**Religion**					
Traditional	19(54.3)	11(31.4)	5(14.3)	35	
Muslim	649(38.4)	381(22.5)	661(39.1)	1691	0.001
Christian	967(34.5)	859(30.6)	978(34.9)	2804	
**Educational status**					
Secondary and above	80(20.2)	124(31.3)	192(48.5)	396	
Primary	185(38.4)	131(27.2)	166(34.4)	482	0.001
Illiterate	1370(37.5)	996(27.3)	1286(35.2)	3652	
**Parity**					
0–1	443(38.4)	325(28.2)	385(33.4)	1153	
2_4	654(36.4)	484(26.9)	660(36.7)	1798	0.091
≥5	538(34.1)	442(28.0)	599(37.9)	1579	
**History of ANC follow up**					
Yes	461(30.5)	492(32.5)	559(37.0)	1512	0.001
No	1174(38.4)	759(25.2)	1085(36.0)	3018	
**Time of death**					
Antepartum	34(41.0)	30(36.1)	19(22.9)	83	
Intrapartum	456(36.8)	360(29.1)	422(34.1)	238	0.019
Post-Partum	1145(35.7)	861(26.8)	1203(37.5)	3209	

### The proportion of assigned cause of death for reviewed maternal death

Women who died due to unanticipated complications of management had a higher proportion of death because of delay three (52.3%) compared to women who were deceased due to other causes of death (36.1%). Women who died due to other obstetrics complications had a higher proportion of death because of delay two (39.1%) compared with the remaining cause of death (27.3%). The proportion of delay one was higher among women who died due to abortive outcomes of pregnancy (43.1%) compared to women who died due to other causes of death (Table 3).

**Table 3:** Distribution of the cause of death by time of death among reviewed maternal death in Ethiopia, 2021.

Cause of death	Delay factors			Total	Significant
	Delay one(n = 1635) (%)	Delay two(n = 1251) (%)	Delay three(n = 1644) (%)		
**Unanticipated complication of management**					
Yes	10(15.4)	21(32.3)	34(52.3)	65	0.001
No	1625(36.4)	1230(27.6)	1610(36.1)	4465	
**Other obstetrics complication**					
Yes	34(24.6)	52(39.1)	47(35.3)	133	0.005
No	1601(36.4)	1199(27.3)	1597(36.3)	4397	
**Abortive pregnancy outcome**					
Yes	44(43.1)	23(22.6)	35(34.3)	102	0.284
No	1591(35.9)	1228(27.7)	1609(36.3)	4428	
**Unknown /undetermined**					
Yes	82(38.3)	74(30.1)	90(36.6)	246	0.564
No	1553(36.3)	1177(27.5)	1554(36.3)	4284	
**Pregnancy-related infection**					
Yes	135(37.0)	93(25.5)	137(37.5)	365	0.634
No	1500(36.0)	1158(27.8)	1507(36.2)	4165	
**Non-obstetrics complication**					
Yes	189(39.6)	133(27.9)	155(32.5)	477	0.137
No	1446(35.7)	1118(27.6)	1489(36.7)	4053	
**Hypertensive disorder of pregnancy**					
Yes	222(35.3)	158(25.1)	249(39.6)	629	0.138
No	1413(36.2)	1093(28.0)	1395(35.8)	3901	
**Obstetric haemorrhage**					
Yes	919(36.6)	697(27.7)	897(35.7)	2513	0.625
No	716(35.5)	554(27.5)	747(37.0)	2017	

### Multilevel multinomial analysis

A multilevel multinomial logistic regression analysis was fitted to assess the three-delay factor related to maternal death. In the multivariate multilevel multinomial regression analysis, both individual level and provincial level variables were associated with delay two and delay three.

#### Factors associated with delay two among reviewed maternal death in Ethiopia

Women’s age, marital status, educational status, place of death, and history of ANC follow-up were among individual-level factors related to delay two. Residence and type of health facility were provincial level factors associated with delay two. Being in an older age group was associated with a higher risk of facing delay two compared to younger age women. Women who died at home and at a health facility had 69% [RRR = 0.31,95%CI:(0.24–0.41)] and 30% [RRR = 0.70,95%CI:(0.55–0.89)] lower risks of encountering delay two, respectively. Women who are married had a 51% [RRR = 1.51, 95%CI: (1.09–2.09)] higher risk of facing delay two compared to their unmarried counterparts. Women with no and primary education had 43% [RRR = 0.57, 95%CI: (0.39–0.89)] and 46% [RRR = 0.54, 95%CI:(0.39–0.73)] lower risk of experiencing delay two, respectively. Women who attend ANC follow-up had a 51% [RRR = 1.51, 95%CI:(1.26–1.81)] higher risk of facing up delay two. Women who were served in secondary-level health facilities had a 43% [RRR = 1.43, 95%CI:(1.09–1.87)] higher risk of encountering delay two. Conversely, women who resided in a rural area had a 26% [RRR = 0.74, 95%CI:(0.56–0.97)] lower risk of facing delay two as compared to women who live in an urban area (Table 4).

**Table 4:** Multilevel multinomial logistic regression analysis in assessing the factors associated with delay factor related to maternal death (both delay two and three).

Variables/Characteristics	Empty model	Model 2^b^		Model 3^c^		Model 4^d^	
		Individual characteristics		Community characteristics		Individual and Community characteristics	
	Delay one^(a)^	Delay two	Delay three	Delay two	Delay three	Delay two	Delay three
		RRR (95%CI)	RRR (95%CI)	RRR (95%CI)	RRR (95%CI)	RRR (95%CI)	RRR (95%CI)
**Age group**							
10_19Y^®^		1	1			1	1
20_29Y		1.20(0.86,1.69)	1.29(0.93,1.78)			1.19(0.85,1.67)	1.25(0.90,1.74)
30_39Y		1.31(0.93,1.85)	1.39(1.00,1.93)			1.30(0.92,1.84)	1.41(1.01,1.97) *
40-49Y		1.63(1.04,2.55) *	1.24(0.79,1.96)			1.62(1.04,2.54) *	1.29(0.82,2.04)
**Place of death**							
On transit ^®^		1	1			1	1
Home		0.31(0.24,0.40) ***	0.16(0.11,0.22) ***			0.31(0.24,0.41) ***	0.17(0.12,0.23) ***
Health facility		0.76(0.61,0.95) *	1.65(1.30,2.08) ***			0.70(0.55,0.89) ***	1.25(0.98,1.60)
**Marital status**							
Unmarried ^®^		1	1			1	1
Married		1.48(1.07,2.05) *	1.27(0.92,1.74)			1.51(1.09,2.09) *	1.26(0.91,1.74)
**Educational status**							
Secondary and above ^®^		1	1			1	1
Illiterate		0.55(0.38,0.79) ***	0.50(0.35,0.72) ***			0.57(0.39,0.82) **	0.53(0.37,0.76) **
Primary		0.53(0.39,0.72) ***	0.47(0.35,0.64) ***			0.54(0.39,0.73) ***	0.47(0.35,0.63) ***
**History of ANC follow-up**							
No^®^		1	1			1	1
Yes		1.59(1.34,1.90) ***	1.26(1.06,1.50) *			1.51(1.26,1.81) ***	1.17(0.98,1.40)
**Time of death**							
Antepartum ^®^		1	1			1	1
Intrapartum		1.07(0.63,1.83)	1.92(1.04,8.25)			1.17(0.68,2.01)	2.33(1.25,4.32) **
Postpartum		1.03(0.61,2.50)	2.34(1.01,16.44)			1.09(0.64,1.84)	2.60(1.42,4.76) **
**Type of health facility**							
Primary health care ^®^				1	1	1	1
Secondary health care				1.47(1.19,1.80) ***	2.63(2.17,3.19) ***	1.24(0.99,1.56)	1.66(1.35,2.05) ***
Tertiary health care				1.67(1.30,2.15) ***	3.82(3.02,4.83) ***	1.43(1.09,1.87) **	2.41(1.88,3.09) ***
**Type of region**							
City administration ^®^				1	1	1	1
Pastoralist region				0.76(0.45,1.31)	0.52(0.31,0.87) *	0.85(0.50,1.44)	0.59(0.35,0.99) *
Agrarian region				0.91(0.58,1.42)	0.80(0.52,1.22)	1.01(0.65,1.56)	0.90(0.59,1.36)
**Residence**							
Urban ^®^				1	1	1	1
Rural				0.63(0.49,0.82) ***	0.64(0.50,0.81) ***	0.74(0.56,0.97) *	0.71(0.55,0.92) **

*P < 0.05,

**P < 0.001,

***P < 0.0001,

^(a)^ Reference for the dependent variable,

^®^ Reference for the category of an independent variable

#### Factors associated with delay three among reviewed maternal death in Ethiopia

Women’s age, educational status, place of death, and time of death were among individual-level factors related to delay three. Residence, type of health facility, and region were provincial factors associated with delay three. Women in the age group 20–24 had 41% [RRR = 1.41, 95%CI: (1.01–1.97)] higher risks of encountering delay three. Women who died at home had an 83% [RRR = 0.17, 95%CI:(0.12–0.23)] lower risk of confronting delay three compared to those who died at a health facility. Women with no education and primary level education had a 47% [RRR = 0.53, 95%CI: (0.37–0.76)] and 53% [RRR = 0.47, 95%CI:(0.35–0.63)] lower risk of facing delay three, respectively. Women who died during intrapartum and postpartum period were associated with 2.33 [RRR = 2.33, 95%CI: (1.25–4.32)] and 2.60 [RRR = 2.66 95%CI: (1.42–4.76)] times higher risk of encountering delay three, respectively as compared to women who died during the antepartum period. Women who were managed in secondary and tertiary level facilities were associated with 1.66 [RRR = 1.66, 95%CI: (1.35–2.05)] and 2.41 [RRR = 2.41, 95%CI: (1.88–3.09)] times higher risk of being challenged by delay three respectively as compared to women who were treated in the primary level of care. Women who live in pastoralist areas had a 41% [RRR = 0.59, 95%CI: (0.35–0.99)] lower risk of confronting delay three as compared to women who live in city administration. Furthermore, women who resided in a rural area had a 29% [RRR = 0.71, 95%CI: (0.55–0.92)] lower risk of facing delay three compared to women who live in an urban area (Table 4).

*Random effect analysis*. As depicted in Table 5, ICC in the null model revealed that about 7% of the variability in delay factor was attributed to differences between provinces. While the highest PCV in the final model revealed that about 45% of the variability of delay factor was explained by both individual and province-level factors. Overall, model 3 was selected as the best-fitted model, due to its low AIC and high log-likelihood (Table 5).

**Table 5:** The random effect model in examining the factors linked to delays relate to maternal death in Ethiopia.

Random effect	Null model^a^	Model_1^b^	Model _2^c^	Model_ 3^d^
**District level variance (SE)**	0.25(0.07)	0.15(0.06)	0.21(0.07)	0.14(0.05)
**P_ values**	<0.001	<0.001	<0.001	<0.001
**ICC (%)**	7%	4.4%	6.0%	4.0%
**Explained variance (PVC) (%)**	Reference	40.0%	16.0%	45.0%
**MOR (95%CI)**	1.60(1.43,1.86)	1.44(1.29,1.69)	1.54(1.37,1.81)	1.42(1.29,1.64)
**Model fit statics**				
**Log-likelihood**	-4883	-4602	-4744	-4555
**AIC**	9766	9218	9496	9130
**BIC**	9770	9230	9502	9147

## Discussion

This assessment is the first comprehensive analysis of MDSR data from 2013 to 2021 that focuses specifically on the magnitude and factors related to delays, which contribute to maternal death. Our key finding revealed that delay one and delay three were major contributors to maternal death in Ethiopia; in addition, the delays were influenced by both individual and provincial level factors.

The magnitude of delay one and delay three were comparable with a study conducted in Ethiopia [25]; however, the magnitude of delay one was much lower compared to studies conducted in Addis Ababa [35], Gamo zone [44], and Malawi [45], while the magnitude of delay three was comparable with a hospital-level study conducted in Ethiopia [46]. The disparity in magnitude could be explained by a variety of reasons such as the data sources, the study population, the study area, and the method of analysis employed. Delay one and delay three were responsible for 72% of maternal death in Ethiopia, hence measures targeting both delays should be devised to substantially reduce maternal death.

The study revealed that the risk of encountering delay two and three was higher in older-aged women compared to younger-age women. This is in line with similar studies done in Nigeria [47], Ghana [48], India [49], and Bangladesh [50]. This could be explained by the high tendency of teenage women (those between 10 to 19 years of age) to hesitate seeking care, due mostly to unwanted and unsupported pregnancy. Those women would prefer home delivery to avoid social exposure making them more vulnerable to delay one as compared to relatively older aged women who had a prior history of giving birth. This has a relationship with the utilization of ANC service, which is commonly influenced by educational status, wealth, and proximity to health facilities [51–53]. Conversely, older women were good at deciding to seek care than younger women and they tend to prefer facility delivery over home delivery. While women encounter numerous challenges hampering them from reaching and receiving care; overall, maternal age is one of the main factors and hence it should be considered in any intervention intended to manage delays related to maternal death.

Place of death, which is believed to be their place of delivery, was negatively associated with delay two and delay three. Women who died at home were less likely to encounter delay two and delay three as compared to women who died during transit. Likewise, women who died at a health facility were less likely to face delay two as compared to women who died during referral. The finding has coherence with studies conducted in Ethiopia (Afar), India, and Nepal [49, 54, 55]. The possible explanation might be due to the expectation that women who deliver at home would fail into the trap of delay in deciding to seek care. Most of the women residing in rural areas would mostly prefer traditional birth attendants due to lack of birth preparedness plans [56–58]. On the other hand, women who died during transit were mostly affected by the unavailability of transportation on their referral from a peripheral health facility [59, 60]. Considering the gap, Ethiopia has established maternity waiting rooms (MWRs), which serve as a waiting area until labor is initiated. The major triggering factors for the establishment of MWRs, among others, were to address barriers to reach to care due to distance, unfavorable seasonal climate, and lack of infrastructure (transportation and effective referral communication) [61]. However, in the Ethiopian context, the utilization of MWRs is affected by the proximity of the mother to a health facility, availability of companion support, and poor awareness about the service [62, 63]. Overall, the finding implied that women who died in different places might encounter varying types of delays based on the delineated circumstance.

This study has demonstrated a statistical significance between marital status and delay two. Married women were more likely to face delay two as compared to unmarried women. This finding was parallel with studies done in Ethiopia (Sidama and Addis Ababa) [64, 65] Nepal [66], Bangladesh [67], India [68] and Myanmar [69]. This might be explained by the positive role husbands play in encouraging women to utilize maternity services. Besides, the husband’s awareness could alert them to take measures timely in addition to availing the necessary resources. This finding implies that male partner involvement could have a positive contribution in reducing the chance of facing delay one, which is a delay in deciding to seek care.

This study has also revealed the association of educational status with delay factors. Women with no education and primary level education were less likely to encounter delay two and three as compared to women who attended school up to secondary and above. Previous studies elsewhere have also demonstrated the correlation between educational status and delay factors related to maternal death [70, 71]. Women’s education enhances health-seeking behavior, and this may lead to early recognition of maternal complications and reduces the risk of facing delay one, which is usually related to delay in deciding to seek care when experiencing an obstetric emergency. Acknowledging the relevance of health education during delivery, Ethiopia initiated a health extension program in 2003 to provide health service and education at a community level, while the implementation and functionality are challenged by the capacity of health posts and the high turnover rate of health extension workers, it has shown a promising stride [72]. Overall, this finding suggested that women’s education status has a significant role in addressing delays related to maternal death.

The study also revealed that history of ANC follow-up has a connection with the delay factor. Women who attended ANC follow-up have a higher chance of encountering delay two than delay one. This is consistent with study findings from Ethiopia [73], Indonesia, and the Philippines [74]. The plausible reason for this could be the fact that women who attended ANC service are more likely to remain vigilant in identifying and intervening to problems in a timely fashion, which has a significant effect on handling delays related to their health-seeking behavior. However, to better achieve the objectives of the ANC service, it should be augmented by better accessibility and availability of health facilities.

The timing of maternal death was also found to be correlated with delay factor. Women who died during the intrapartum and postpartum period were more likely to face delay three than women who died during the antepartum period. Previous studies elsewhere have also disclosed similar findings, i.e., delay three plays a decisive role in determining the timing of maternal death [75, 76]. This might be directly related to the absence of essential medical commodities, trained health professionals, and well-equipped facilities.

The type of health facility, where the woman visited for an obstetric complication, has also shown an association with delay factors. Women who were treated at a tertiary level facility have a higher chance of facing delay two and delay three compared to other levels of care. Besides, women who were managed in a secondary level facility were more likely to encounter delay three. This finding was congruent with studies done in Uganda [77], Tanzania [78] and India [79, 80]. This could be explained by the poor handling of obstetrics emergencies at lower-level facilities, which in turn, result in overwhelming the managing capacity of the next higher-level facility due to unreasonable referral. Moreover, the presence of poor referral linkage has resulted in a delayed referral and paved the way for multiple referrals, which has a negative consequence on the outcome of the mother. The introduction of mentorship and coaching is one of the new approaches implemented in Sub-Sahara African countries to avert the challenge of poor service provision at lower-level facilities. The mentorship practice allows close observation of lower facilities by availing trained health professionals and equipment to handle obstetrics emergencies [81]. Overall, this finding suggested the need to evaluate the facility-level readiness on a timely basis based on health facilities’ capacity to manage obstetric complications.

The study has identified the presence of regional variation in the delay factors that contributed to maternal death. Women who lived in pastoralist areas have demonstrated a lower risk of facing delay three compared to women who reside in metropolitan cities. Besides women who live in rural areas have shown a lower risk of encountering delays two and three compared with their counterparts who live in urban areas. The finding was comparable with studies done elsewhere [24, 82–84], which revealed the presence of inequality in maternal service utilization based on residence and region. The likely explanation for this could be the fact that women who reside in rural areas and pastoralist regions have limited information on maternity services; in addition, this could also be due to the challenge residents face in accessing health facilities within a reasonable distance and time. The combination of all these factors hinders women from utilizing services and negatively affects their health-seeking behavior. Overall, the finding implies the need for narrowing down the disparity between regions in service availability and utilization.

The study has the following limitations that need to be admitted. First, almost all death review was conducted in public facilities with limited involvement of private facilities, and this could affect the inclusiveness of the study. Secondly, variation among regions in the implementation of the MDSR system may affect the representativeness of the study. Finally, the available variables in the dataset may not be adequate in explaining factors related to delays.

## Conclusion

Overall, this study was guided by two research questions, aiming to investigate the magnitude; and factors that determine delays related to maternal death. The study adds knowledge on the impact of delay factors in maternal death in Ethiopia. It also pinpoints a critical area of focus to contend with delay factors at both individual (maternal age, marital status, place of death, timing of death, and education status) and provincial (residence, type region, and facility) levels. With the above-mentioned limitation in mind, some conclusions and recommendations can be drawn from the results obtained. First, improving the health-seeking behavior of the community through various channels such as using media campaigns, frontline health workers (health extension workers), and routine ANC care is crucial. Besides, interventions aimed at improving the engagement of younger-aged women and male companions should be the other front to reduce death contributed by delay one. Second, more emphasis and attention should be given to the crucial time of pregnancy, especially during the intrapartum and postpartum period, by improving the accessibility to maternity service by maximizing the utilization of MWRs to handle maternal death due to delay two. Third, facility-level readiness should be ameliorated in terms of manpower and equipment along with smoothening the referral system among facilities by giving special emphasis on the lower-level facilities to address delay three.
